# Effect of obicetrapib, a potent cholesteryl ester transfer protein inhibitor, on p-tau217 levels in patients with cardiovascular disease

**DOI:** 10.1016/j.tjpad.2025.100394

**Published:** 2025-10-17

**Authors:** Michael H Davidson, Michael Szarek, Philip Scheltens, Everard Vijverberg, Andrew Hsieh, Marc Ditmarsch, Douglas Kling, Danielle Curcio, Stephen J Nicholls, Kausik K Ray, Jeffrey L. Cummings, John JP Kastelein

**Affiliations:** a NewAmsterdam Pharma, Amsterdam, the Netherlands; b University of Colorado Anschutz Medical Campus and CPC Clinical Research, Aurora, CO, USA; c Mount Sinai Fuster Heart Hospital, Icahn School of Medicine at Mount Sinai, NY, NY, USA; d State University of New York Downstate School of Public Health, Brooklyn, NY, USA; e Alzheimer Center Amsterdam, Neurologie, Vrije Universiteit Amsterdam, Amsterdam University Medical Center, Amsterdam, the Netherlands; f EQT Life Sciences, Amsterdam, the Netherlands; g Amsterdam Neuroscience, Neurodegeneration, Amsterdam UMC location VUMC, Amsterdam, the Netherlands; h Victorian Heart Institute, Monash University, Australia; i Imperial College London School of Public Health, London, United Kingdom; j Chambers-Grundy Center for Transformative Neuroscience, Department of Brain Health, Kirk Kerkorian School of Medicine, University of Nevada, Las Vegas, Las Vegas, NV, USA

**Keywords:** Alzheimer's disease prevention, APOE4, CETP inhibitor, Phosphorylated tau-217, Obicetrapib

## Abstract

**Background:**

Cholesteryl ester transfer protein (CETP) inhibition reduces low density lipoprotein-cholesterol (LDL-C) while simultaneously increasing high density lipoprotein-cholesterol (HDL-C) levels and improving HDL-particle functionality. These lipoprotein modifications may provide a novel pathway for Alzheimer disease (AD) prevention through effects on lipid modulation, antioxidant activity, and neuro-inflammation. This approach could prove particularly beneficial for APOE4 carriers, who face elevated risks for both AD and atherosclerotic cardiovascular disease (ASCVD).

**Objectives:**

To examine the effects of obicetrapib, an oral CETP inhibitor, on biomarker changes indicative of AD pathology among patients with ASCVD

**Design:**

This was a pre-specified substudy of the BROADWAY trial, a phase 3, double-blind, placebo-controlled pivotal registration trial to evaluate the LDL-C lowering efficacy of obicetrapib in adult patients with established ASCVD and/or heterozygous familial hypercholesterolemia (HeFH), whose LDL-C was not adequately controlled, despite being on maximally tolerated lipid-lowering therapy.

**Setting:**

The trial was conducted across 188 sites in China, Europe, Japan, and the United States. Participants were recruited from cardiology clinics and lipid specialty centers from 2021 to 2024.

**Participants:**

Participants with ASCVD in BROADWAY who had known ApoE status and phosphorylated tau-217 (p-tau217) measured at baseline and 12 months.

**Intervention:**

Participants in BROADWAY were randomized 2:1 to receive oral obicetrapib 10 mg daily or placebo for 12 months.

**Measurements:**

AD plasma biomarkers were measured at baseline and 12 months using standardized SIMOA assays. The key outcome measure of interest was change in plasma p-tau217 from baseline to 12 months. Other outcome measures included changes in p-tau217/(Aβ42:40), p-tau181, glial fibrillary acidic protein (GFAP), and neurofilament light chain (NfL).

**Results:**

The analysis population consisted of 1535 (61 %) of the 2530 BROADWAY participants. Median age was 67 years and 67.0 % were male. Baseline p-tau217 levels varied significantly by ApoE subgroups, with ApoE4 carriers generally having higher concentrations and ApoE4/E4 participants exhibiting the highest median concentration (0.56 pg/mL). Obicetrapib significantly attenuated p-tau217 increases compared to placebo (adjusted mean 2.09 % vs 4.94 %; *P* = 0.025). Treatment differences were most pronounced in ApoE4 carriers, where adjusted mean increases were 1.92 % and 6.91 %, for obicetrapib and placebo, respectively (*P* = 0.041). Furthermore, among ApoE4/E4 participants, there was a 7.81 % adjusted mean decrease in p-tau217 with obicetrapib compared to a 12.67 % increase with placebo, representing a 20.48 % treatment difference (*P* = 0.010). Positive trends were observed across secondary biomarkers, with obicetrapib also significantly limiting increases in the p-tau217/Aβ42:40 ratio compared to placebo (2.51 % vs 6.55 %; *P* = 0.004). In addition, among ApoE4/E4 participants, obicetrapib demonstrated significant effects on GFAP (-6.39 % vs +8.85 %; *P* = 0.006) and NfL (-10.49 % vs +6.82 %; *P* = 0.020). Strong correlations were observed between end-of-study obicetrapib plasma concentrations and biomarker improvements (*r*=-0.64), suggesting CETP inhibition as a potential mechanism, although other drug effects may also contribute to these changes.

**Conclusions:**

Obicetrapib significantly slowed AD biomarker progression over 12 months in participants with ASCVD, with the greatest effects in ApoE4 carriers. Among ApoE4/E4 participants, obicetrapib reduced p-tau217 levels by a placebo-adjusted 20.48 % and demonstrated consistent effects across multiple AD biomarkers. These findings represent the first demonstration of an oral intervention capable of reducing both beta-amyloid and tau pathology biomarkers in ApoE4 carriers, offering a potential preventive strategy for this high-risk population who currently have no effective prevention options. Future research will need to establish whether these biomarker changes translate to clinical benefits in dedicated AD prevention trials.

**Trial Registration:**

ClinicalTrials.gov Identifier: NCT05142722.

## 1 Introduction

Dysregulation of lipid metabolism in the brain plays a central role in the early development and progression of Alzheimer’s disease (AD) [1]. Cholesterol and phospholipids represent critical membrane components in neurons and synapses, influencing essential cellular processes including signaling pathways, inflammatory responses, and protein trafficking [2] The lipid dysregulation hypothesis suggests that when lipid homeostasis becomes disrupted, it directly contributes to AD's characteristic pathological features: amyloid-beta (Aβ) plaques, tau tangles, and progressive neuronal loss [3].

The relationship between lipid dysregulation and AD centers largely on apolipoprotein E (APOE) status. The APOE ε4 (E4) allele demonstrates reduced efficiency in transporting cholesterol from astrocytes to neurons and clearing cholesterol from various cell types, when compared to E3 or E2 variants [4]. These functional deficiencies result in lipid accumulation within specific brain regions and enhanced Aβ aggregation [5]. Moreover, APOE4-induced cholesterol dysregulation produces cell-specific effects that disrupt neuronal synaptic function, alter astrocytic glucose metabolism, promote microglial inflammatory responses, and impair oligodendrocyte remyelination, all processes that contribute significantly to AD pathogenesis. In peripheral circulation, APOE4 carriers exhibit a distinctive lipid profile characterized by elevated LDL-C, apolipoprotein B, and Lp(a), as well as reduced HDL-C. These alterations increase cardiovascular disease risk and create a metabolic environment that promotes AD pathogenesis [[6], [7], [8], [9]].

Cholesteryl ester transfer protein (CETP) occupies a central position in systemic lipid metabolism by facilitating cholesteryl ester transfer from HDL to LDL [10]. This transfer activity reduces HDL-C levels while increasing LDL-C content, effectively shifting the balance toward a more atherogenic and pro-inflammatory lipid profile. Importantly, CETP is also expressed in brain tissue, particularly in astrocytes, where it may directly influence central nervous system (CNS) cholesterol homeostasis. Evidence from transgenic mouse models demonstrates that increased CETP activity substantially impacts brain cholesterol levels, with human CETP transgenic mice showing up to 22 % higher brain cholesterol content when challenged with dietary cholesterol. This underscores CETP's direct role in modulating brain lipid metabolism [[11], [12], [13]].

Evidence from multiple studies have demonstrated that CETP genetic variants influence cognitive outcomes, with stronger effects in APOE4 carriers [14]. In a prospective cohort study, individuals homozygous for the low CETP activity V405 polymorphism demonstrated slower memory decline and a 72 % reduction in dementia risk (HR 0.28; 95 % CI, 0.10–0.85) [15]. Other work has found that this variant buffers memory decline in APOE4 carriers through a dose-dependent mechanism, APOE4 carriers with two copies of the V405 variant showed memory decline rates comparable to those without APOE4 [16]. Large-scale population studies confirmed that the valine allele slows cognitive decline over time [17]. CETP promoter variants also modify Alzheimer's risk in APOE4 carriers, with certain genotypes conferring substantial protection [18]. Together, these findings suggest CETP regulates brain cholesterol metabolism in ways that may protect against cognitive decline, particularly for those carrying APOE4. These genetic findings receive further support from recent Mendelian randomization analyses, demonstrating that genetically determined lower CETP concentration is associated with higher total brain volume and decreased risk of Lewy body and Parkinson's dementia, with greater effect size in APOE4 carriers [19].

Plasma biomarkers are now available to assess the effects of interventions on AD pathobiology. P-tau 217 and p-tau 181 correlate with amyloid-beta plaque pathology; Aβ42:Aβ40 ratio reflects amyloid pathology, declining as Aβ42 is trapped in the brain plaques; glial fibrillary acidic protein (GFAP) measures astroglial activation associated with inflammation; and neurofilament light chain (NfL) reflects axonal degeneration [20]. Biomarker changes in AD are evident in the preclinical period of AD when brain changes are present but cognitive decline is not yet present making them appropriate as foundational measures for AD prevention therapies [21].

Emerging data in patients with cardiovascular disease has demonstrated undetected cognitive dysfunction within this population. Recent evaluation of individuals with ASCVD demonstrates that approximately one-third exhibit performance deficits on standardized cognitive instruments, despite the absence of formal dementia or MCI diagnoses. Biomarker analyses further indicate that elevated AD-related proteins, particularly p-tau217, occur in over half of cardiovascular patients tested. This prevalence substantially exceeds the general population estimates and was demonstrated despite regular medical monitoring [22]. These observations provide support for developing therapeutic interventions targeting both vascular and neurodegenerative mechanisms, particularly as ASCVD patients are subjected to regular follow-up yet experience under-recognition of brain pathology.

Obicetrapib is an oral CETP inhibitor that has undergone phase 3 testing in more than 3200 patients and modifies lipids with a unremarkable safety profile [23,24]. In an initial proof-of-concept (POC) study with 13 APOE4 positive AD patients with mild cognitive impairment (MCI), obicetrapib decreased plasma and cerebrospinal fluid levels (CSF) of 24S- and 27-hydroxycholesterol, increased levels of critical lipophilic antioxidants and carotenoids, and stabilized key biomarkers associated with AD. These results suggested potential disease-modifying effects of obicetrapib (NCT05161715) [25].

We present findings from a prespecified substudy of the BROADWAY trial examining obicetrapib's effects on p-tau217 and other AD biomarkers in patients with ASCVD. In preclinical AD, p-tau217 accurately identifies individuals with amyloid pathology and predicts progression from MCI to AD dementia with high accuracy [26,27].

We hypothesized that obicetrapib's effects on lipid metabolism, including HDL-C elevation and improved HDL particle functionality, LDL-C reduction, anti-inflammatory actions, and vascular benefits, would attenuate p-tau217 progression. We further anticipated, based on genetic evidence, that these effects would be more evident among APOE4 carriers.

## 2 Methods

### 2.1 Study design and participants

The BROADWAY trial was conducted across 188 sites in China, Europe, Japan, and the United States. Participants were recruited from cardiology clinics and lipid specialty centers, with eligibility requiring documented atherosclerotic cardiovascular disease (ASCVD) and/or clinical or genetic diagnosis of heterozygous familial hypercholesterolemia (HeFH). All participants had LDL-C levels that remained inadequately controlled despite treatment with maximally tolerated doses of lipid lowering therapies [23].

BROADWAY had demonstrated that obicetrapib 10 mg had significantly reduced LDL-C compared to placebo. At day 84, LDL-C decreased by 29.9 % with obicetrapib versus an increase of 2.7 % with placebo, yielding a difference of 32.6 % (*P* < 0.001). Treatment with obicetrapib also significantly increased HDL-C by 136.3 % and lowered lipoprotein(a) by 33.5 %. Safety profiles were comparable between groups. Complete details of the BROADWAY recruitment strategy, inclusion/exclusion criteria, and site characteristics are reported in the primary publication [23,28]. In the context of these lipid changes, the current substudy examined whether obicetrapib treatment additionally affects biomarkers related to Alzheimer's progression.

This pre-specified substudy utilized stored plasma samples from participants in the BROADWAY study. The BROADWAY protocol incorporated provisions for collecting and storing additional blood samples to enable biomarker testing. Given emerging evidence that links lipid metabolism with AD pathobiology and neurodegeneration, particularly among APOE4 carriers, and following the successful completion of our POC study demonstrating obicetrapib's effects on cholesterol metabolites and antioxidant levels in AD patients, as well as recommendations from our AD experts. AD biomarker assessment was identified as a key area of investigation. A supplemental statistical analysis plan was developed and finalized to guide the analysis of these outcome measures.

For inclusion in the present AD biomarker analysis population, participants with known ApoE status had to have baseline plasma p-tau217 concentrations above the lower limit of quantification (LLQ; 0.06 pg/mL) and available end-of-study p-tau217 measurements. A priori testing was not permitted in certain countries due to ethical and regulatory constraints. In the Netherlands, the informed consent form limited the use of data and samples to objectives and analyses explicitly defined in the study protocol, thereby excluding additional analyses such as those related to potential future evaluation of dyslipidemia and/or CV risk that were only broadly referenced in the protocol. In China, regulatory restrictions prevent the export of biological samples, making them unavailable for analysis.

The study was conducted in accordance with the Declaration of Helsinki and Good Clinical Practice guidelines. All participants provided written informed consent, and institutional review boards at participating centers approved the protocol.

### 2.2 Sample collection and biomarker measurements

Blood samples for AD biomarker assessment were collected at baseline (Visit 2) and at 12 months (Visit 7) as part of the stored sample collection protocol. Sample handling required immediate placement on wet ice prior to centrifugation at 4 °C. When cooled centrifuges were unavailable, centrifuge racks were pre-cooled for 30 min at −20 °C before centrifugation. These standardized conditions were critical for preserving sample integrity, especially for amyloid-beta peptides which degrade readily at ambient temperature.

All biomarker analyses were conducted at a single centralized laboratory (Medpace Reference Lab, Cincinnati, OH) using validated assays on the Single-Molecular Array (SIMOA) HD-X Analyzer platform. Plasma p-tau217 was measured using the ALZpath SIMOA pTau-217 v2 assay, with within-run imprecision of 3.8–4.7 % coefficient of variation (CV) and between-run imprecision of 7.3–9.3 % CV. Plasma Aβ40, Aβ42, GFAP, and NfL were measured simultaneously using the SIMOA Neurology 4-Plex E Advantage Kit, with between-run imprecision ranging from 6.4–14.0 % CV across analytes. Plasma p-tau181 was quantified using the SIMOA pTau-181 Advantage V2.1 assay. Results for amyloid-beta were expressed as the Aβ42:Aβ40 ratio to account for potential degradation effects. All assays utilized EDTA-plasma samples. Given the sample size, multiple reagent lots were required, with bridging studies performed between lots to ensure assay consistency. Laboratory personnel remained blinded to treatment assignment throughout the analysis process, with samples distributed across reagent lots regardless of treatment allocation.

ApoE status was determined using ApoE isoform phenotyping, with participants classified into standard allele combination subtypes: ApoE2/E2, ApoE2/E3, ApoE3/E3, ApoE2/E4, ApoE3/E4, or ApoE4/E4.

### 2.3 Outcomes

The primary outcome measure for this substudy was the change in plasma p-tau217 concentration from baseline to 12 months. Additional outcomes included changes in the p-tau217/(Aβ42:Aβ40) ratio, p-tau181, GFAP, and NfL from baseline to 12 months. These biomarkers were selected based on their established roles in AD pathogenesis.

P-tau217 outcomes were assessed in the overall analysis population and separately for all ApoE4 carriers (E2/E4, E3/E4, E4/E4), ApoE carriers excluding ApoE2/E4, ApoE3/E4, and ApoE4/E4. Other biomarkers were assessed overall and separately for ApoE4 carriers (excluding ApoE2/E4), ApoE3/E4, and ApoE4/E4. Additional analyses examined correlations between changes in AD biomarkers and concurrent changes in lipid parameters, as well as associations with achieved obicetrapib plasma concentrations.

### 2.4 Statistical analysis

Baseline characteristics were summarized using median (Q1, Q3) for continuous variables and n ( %) for categorical variables. Comparisons between ApoE subgroups were performed using Kruskal-Wallis tests for continuous variables and chi-square tests for categorical variables.

Treatment group differences in absolute and percent changes in AD biomarkers from baseline to end of study were analyzed using robust regression with M estimation to account for outliers. Treatment group means and 95 % confidence intervals were estimated both unadjusted and adjusted for mean-centered baseline biomarker values and mean-centered age. Loess curves were used to examine predicted changes in p-tau217 as functions of age and baseline p-tau217 concentrations, derived from robust regression models that included terms for treatment group, mean-centered baseline p-tau217, mean-centered age, and relevant interaction terms. Adjustment for baseline biomarker values accounts for any baseline imbalances between treatment groups, ensuring that any post-baseline treatment differences are not confounded by differences in baseline distributions.

Among participants with baseline p-tau217 concentrations below 0.42 pg/mL, the likelihood of exceeding this threshold at end of study was compared between treatment groups using logistic regression with baseline p-tau217 and age as covariates.

Relationships between end-of-study obicetrapib concentrations, time-averaged achieved lipid concentrations, and absolute changes in p-tau217 were assessed using Spearman correlation coefficients.

Two-tailed P-values less than 0.05 were considered statistically significant, with no adjustment for multiplicity. All analyses were performed using SAS version 9.4 (SAS Institute) by an independent academic statistician (M.S.) who had access to the complete dataset.

## 3 Results

### 3.1 Study population and baseline characteristics

**Fig. 1:**
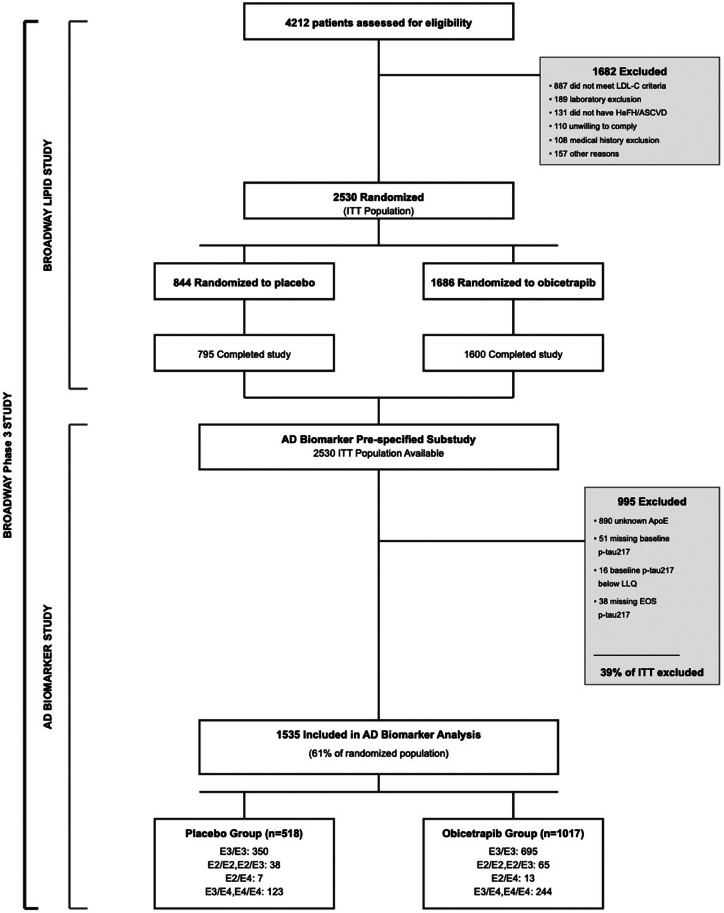
CONSORT Flow Diagram.

Of the 2530 randomized participants, 1535 met the criteria for inclusion in analysis population. Exclusions included participants with unknown ApoE status (*n* = 890), missing baseline p-tau217 (*n* = 51), baseline p-tau217 below LLQ (*n* = 16), and missing end-of-study p-tau217 (*n* = 38). The ApoE allele combination subgroups included 1045 participants who were ApoE3/E3, 103 who were ApoE2/E2 or ApoE2/E3, 20 who were ApoE2/E4, 338 who were ApoE3/E4, and 29 who were ApoE4/E4 (Fig. 1).

Baseline characteristics of the analysis population are summarized in Table 1 and **eTable 1**. Overall, the median age was 67 years, 67.0 % were male, and 84.5 % were Caucasian. The distributions of age (*p* = 0.12), sex (*p* = 0.45), diabetes (*p* = 0.91). hypertension (*p* = 0.10), and established ASCVD (*p* = 0.89) did not significantly differ across subgroups. Racial composition did differ across subgroups (*p* = 0.0011), with higher percentages of Black participants observed among those with at least one ApoE2 allele (i.e., ApoE2/E2, ApoE2/E3, and ApoE2/E4).

**Table 1:** Baseline characteristics.

	E3/E3		E2/E2, E2/E3		E2/E4		E3/E4			E4/E4			P-value*
	Obicetrapib (*n* = 695)	Placebo (*n* = 350)	Obicetrapib (*n* = 65)	Placebo (*n* = 38)	Obicetrapib (*n* = 13)	Placebo (*n* = 7)	Obicetrapib (*n* = 225)	Placebo (*n* = 113)	Obicetrapib (*n* = 19)		Placebo (*n* = 10)		
Age, years	67 (61, 73)	68 (61, 73)	70 (60, 74)	67 (58, 72)	61 (52, 69)	63 (52, 69)	67 (60, 72)	66 (59, 72)	68 (55, 72)		69 (66, 71)	0.12	
Female sex	225 (32.4)	112 (32.0)	21 (32.3)	16 (42.1)	6 (46.2)	2 (28.6)	82 (36.4)	36 (31.9)	5 (26.3)		1 (10.0)	0.45	
Race												0.0011	
White	585 (84.2)	304 (86.9)	54 (83.1)	30 (78.9)	9 (69.2)	7 (100)	184 (81.8)	100 (88.5)	14 (73.7)		10 (100)		
Asian	69 (9.9)	31 (8.9)	3 (4.6)	2 (5.3)	0	0	19 (8.4)	6 (5.3)	3 (15.8)		0		
Black	31 (4.5)	11 (3.1)	8 (12.3)	6 (15.8)	4 (30.8)	0	19 (18.4)	5 (4.4)	2 (10.5)		0		
Other	10 (1.4)	4 (1.1)	0	0	0	0	3 (1.3)	2 (1.8)	0		0		
Medical History													
Diabetes	252 (36.3)	142 (40.6)	28 (43.1)	13 (34.2)	5 (38.5)	3 (42.9)	84 (37.3)	40 (35.4)	7 (36.8)		6 (60.0)	0.91	
Hypertension	604 (86.9)	297 (84.9)	57 (87.7)	30 (78.9)	12 (92.3)	6 (85.7)	176 (78.2)	95 (84.1)	14 (73.7)		10 (100)	0.10	
ASCVD	611 (87.9)	296 (84.6)	61 (93.8)	32 (84.2)	11 (84.6)	6 (85.7)	193 (85.8)	102 (90.3)	15 (78.9)		10 (100)	0.89	
AD Biomarkers													
p-tau217, pg/mL	0.40 (0.29, 0.54)	0.39 (0.28, 0.58)	0.39 (0.32, 0.46)	0.36 (0.24, 0.49)	0.40 (0.30, 0.48)	0.49 (0.34, 0.54)	0.50 (0.34, 0.72)	0.43 (0.33, 0.70)	0.56 (0.43, 0.91)		0.57 (0.37, 0.73)	<0.0001	
AB42/40	0.055 (0.049, 0.063)	0.056 (0.049, 0.061)	0.057 (0.052, 0.063)	0.056 (0.050, 0.062)	0.057 (0.051, 0.063)	0.055 (0.040, 0.068)	0.053 (0.047, 0.060)	0.052 (0.046, 0.059)	0.050 (0.044, 0.056)		0.051 (0.049, 0.053)	0.0007	
p-tau217/(AB42/40)	6.95 (5.08, 10.34)	6.97 (5.01, 10.75)	6.64 (5.34, 8.69)	5.97 (3.87, 8.21)	6.13 (5.34, 11.31)	8.52 (7.70, 9.99)	9.15 (6.48,14.53)	8.03 (6.13, 15.04)	13.38 (8.44, 16.34)		11.15 (6.37, 14.22)	<0.0001	
GFAP, pg/mL	93.5 (65.9, 129.0)	90.3 (64.3, 124.0)	91.1 (66.6, 161.0)	99.7 (71.1, 126.0)	84.8 (47.6, 100.0)	104.0 (75.1, 113.0)	102.0 (67.8, 151.0)	95.4 (69.4, 135.0)	124.0 (57.5, 178.0)		97.7 (60.5, 146.0)	0.09	
NFL, pg/mL	17.3 (13.0, 25.4)	18.6 (13.1, 26.6)	17.4 (12.8, 26.0)	17.2 (11.9, 23.3)	13.5 (10.0, 15.7)	25.0 (10.8, 30.2)	17.1 (12.6, 25.9)	17.8 (12.7, 28.6)	16.1 (11.7, 19.3)		17.6 (12.4, 26.2)	0.45	
p-tau181, pg/mL	18.7 (14.8, 25.0)	19.1 (14.8, 24.9)	20.0 (15.5, 24.1)	14.6 (13.5, 24.1)	20.1 (16.7, 22.8)	18.3 (15.8, 21.2)	20.2 (16.6, 25.8)	19.3 (16.3, 24.4)	21.2 (16.6, 28.2)		18.9 (15.2, 23.2)	0.0370	

Note: values in table are n ( %) or median (Q1, Q3). ASCVD = atherosclerotic cardiovascular disease.

⁎ P-values for comparisons between ApoE status (pooled across treatment groups) by Kruskal-Wallis or chi-square tests.

**eTable 2** summarizes baseline characteristics for those included in the analysis population versus those in the BROADWAY population that were excluded. The baseline characteristics of the analysis population were comparable with those that were excluded, with similar distributions of age (median 65 years), sex (66 % male), and cardiovascular risk factors. Lipids and lipoproteins differed between the groups, reflecting less high intensity statin use among the excluded participants. Overall, these results suggest that participants who were excluded due to unknown ApoE status or other reasons did not introduce selection bias.

Baseline AD biomarker concentrations differed by ApoE subgroups. The clearest pattern was observed for p-tau217 levels (*p* < 0.0001), where ApoE3/E3 (median 0.39 pg/mL) and ApoE2/E2 or ApoE2/E3 participants (0.38 pg/mL) had the lowest concentrations. Participants who were ApoE2/E4 or ApoE3/E4 had higher concentrations (0.45 and 0.46 pg/mL, respectively), while the highest p-tau217 concentrations were observed among those who were ApoE4/E4 (0.56 pg/mL).

This ApoE-dependent pattern was also apparent in baseline concentrations of other biomarkers. The Aβ42:40 ratio and the p-tau217/(Aβ42:40) ratio varied across subgroups (*p* = 0.0007 and <0.0001, respectively). GFAP levels varied but did not reach statistical significance (*p* = 0.09), while NfL concentrations were similar across ApoE subgroups (*p* = 0.45). P-tau181 levels showed modest but statistically significant differences across similar ApoE subgroups (*p* = 0.037).

### 3.2 Treatment effects on AD biomarkers

Over the 12-month treatment period, obicetrapib demonstrated significant effects on multiple AD biomarkers, with the most pronounced effects observed in ApoE4 carriers.

### 3.3 P-tau217

P-tau217 results are summarized in Table 2. In the overall analysis population (*n* = 1535), obicetrapib significantly attenuated p-tau217 progression compared to placebo: mean (95 % CI) adjusted percent change of 2.09 % (0.65 to 3.54) versus 4.94 % (2.92 to 6.96), representing a 2.84 % reduction from placebo (*p* = 0.025). Similarly significant results were observed for absolute change.

**Table 2:** Change in p-tau217 from baseline to end of study.

	Unadjusted			Adjusted*		
	Obicetrapib	Placebo	P-value	Obicetrapib	Placebo	P-value
All Participants						
Absolute Change, pg/mL	0.007 (0.001, 0.013)	0.025 (0.016, 0.034	0.0010	0.004 (−0.003, 0.010)	0.020 (0.012, 0.029)	0.0022
Percent Change, %	2.11 (0.66, 3.56)	5.15 (3.12, 7.18)	0.0171	2.09 (0.65, 3.54)	4.94 (2.92, 6.96)	0.0247
E3/E3						
Absolute Change, pg/mL	0.008 (0, 0.152)	0.020 (0.010, 0.031)	0.05	0.004 (−0.003, 0.012)	0.016 (0.005, 0.026)	0.08
Percent Change, %	2.03 (0.24, 3.82)	3.75 (1.22, 6.28)	0.28	1.70 (−0.09, 3.49)	3.32 (0.81, 5.83)	0.30
E2/E2, E2/E3						
Absolute Change, pg/mL	0.025 (0.003, 0.046)	0.047 (0.020, 0.075)	0.20	0.052 (0.033, 0.071)	0.083 (0.058, 0.109)	0.05
Percent Change, %	5.59 (0.60, 10.57)	11.85 (5.33, 18.37)	0.13	5.05 (−0.08, 10.19)	10.58 (3.73, 17.42)	0.20
E2/E4, E3/E4, E4/E4						
Absolute Change, pg/mL	0.001 (−0.013, 0.015)	0.033 (0.013, 0.053)	0.0104	0.001 (−0.013, 0.015)	0.027 (0.008, 0.047)	0.0283
Percent Change, %	1.47 (−1.31, 4.24)	6.63 (2.72, 10.53)	0.0347	1.92 (−0.95, 4.79)	6.91 (2.98, 10.85)	0.0414
E3/E4, E4/E4						
Absolute Change, pg/mL	−0.001 (−0.015, 0.014)	0.035 (0.014, 0.055)	0.0067	0 (−0.015, 0.014)	0.029 (0.008, 0.049)	0.0215
Percent Change, %	1.00 (−1.82, 3.82)	6.93 (2.96, 10.90)	0.0171	1.45 (−1.47, 4.38)	7.19 (3.18, 11.20)	0.0216
E3/E4						
Absolute Change, pg/mL	0.004 (−0.012, 0.019)	0.032 (0.010, 0.054)	0.0360	0.002 (−0.013, 0.017)	0.025 (0.004, 0.046)	0.07
Percent Change, %	1.68 (−1.32, 4.68)	6.35 (2.11, 10.58)	0.08	2.10 (−1.00, 5.20)	6.70 (2.15, 10.99)	0.08
E4/E4						
Absolute Change, pg/mL	−0.034 (−0.090, 0.021)	0.060 (−0.016, 0.136)	0.0493	−0.053 (−0123, 0.017)	0.074 (−0.005, 0.154)	0.0174
Percent Change, %	−4.71 (−12.67, 3.24)	12.17 (1.20, 23.13)	0.0146	−7.81 (−18.21, 2.60)	12.67 (0.85, 24.49)	0.0102

The treatment effects on percent change varied across ApoE subgroups. Those who were ApoE3/E3 showed modest differences with obicetrapib [adjusted mean (95 % CI) = 1.70 % (−0.09 to 3.49)] compared to placebo [3.32 % (0.81 to 5.83); *p* = 0.30], while the ApoE2/E2 or ApoE2/E3 subgroup demonstrated larger increases that were not significantly different between treatment groups: obicetrapib [5.05 % (−0.08 to 10.19)] versus placebo [10.58 % (3.73 to 17.42); *p* = 0.20].

Of note, the most pronounced treatment group differences in percent change were observed in ApoE4 carriers. When all ApoE4 carriers were analyzed together, obicetrapib treatment yielded a significantly smaller increase [adjusted mean (95 % CI) = 1.92 % (−0.95 to 4.79)] compared to placebo (6.91 %; 95 % CI, 2.98 to 10.85), or a 4.99 % difference in means (*p* = 0.041). When restricting ApoE4 carriers to ApoE3/E4 or ApoE4/E4, the corresponding changes were 1.45 % (−1.47, 4.38) and 7.19 % (3.18, 11.20), respectively (*p* = 0.022). Furthermore, among ApoE4/E4 participants, there was a 7.81 % decrease in the obicetrapib group and a 12.67 % increase in the placebo group, yielding a 20.48 % difference in means (*p* = 0.010).

**Fig. 2:**
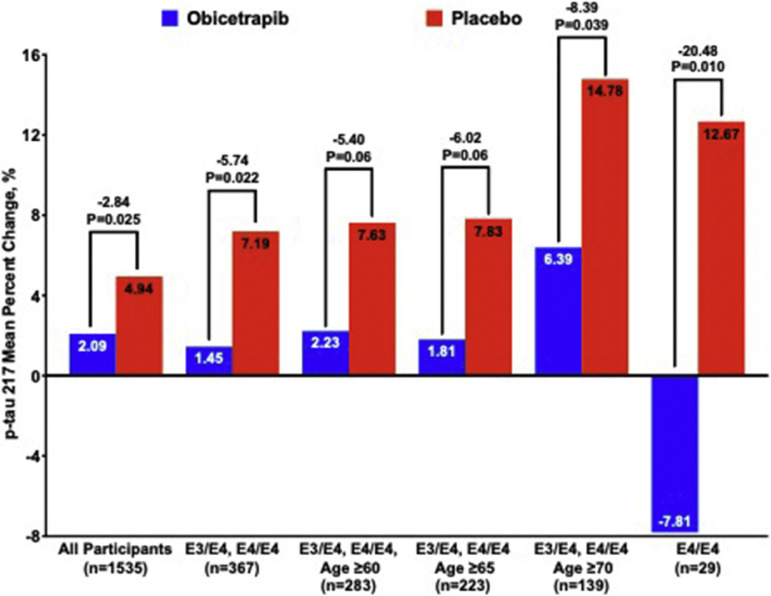
**Percent Change in p-tau217 by Subgroups with Progressively Greater Risk of AD Pathology.** Mean percent changes for each treatment group are from robust regression models with terms for treatment group, mean-centered baseline p-tau217, and mean-centered age.

Fig. 2 illustrates treatment effects on p-tau217 percent change across subgroups with progressively greater risk of AD pathology, demonstrating that both the placebo group deterioration and obicetrapib effects tended to increase with higher pathological risk. Specifically, age-stratified analyses of ApoE3/E4 and ApoE4/E4 participants revealed consistent patterns across different age thresholds. Among ApoE3/E4 or ApoE4/E4 participants aged ≥60 years (*n* = 283), obicetrapib resulted in a 2.23 % adjusted mean increase versus 7.63 % with placebo, yielding a 5.40 % difference (*p* = 0.06). Among those aged ≥65 years (*n* = 223), the adjusted mean increases were 1.81 % versus 7.83 %, representing a 6.02 % difference (*p* = 0.06). Finally, among those aged ≥70 years (*n* = 139), obicetrapib yielded a 6.39 % adjusted mean increase compared to 14.78 % with placebo, translating to a 8.39 % difference (*p* = 0.039).

### 3.4 Relationships between age, baseline P-tau217, and P-tau217 change

**Fig. 3:**
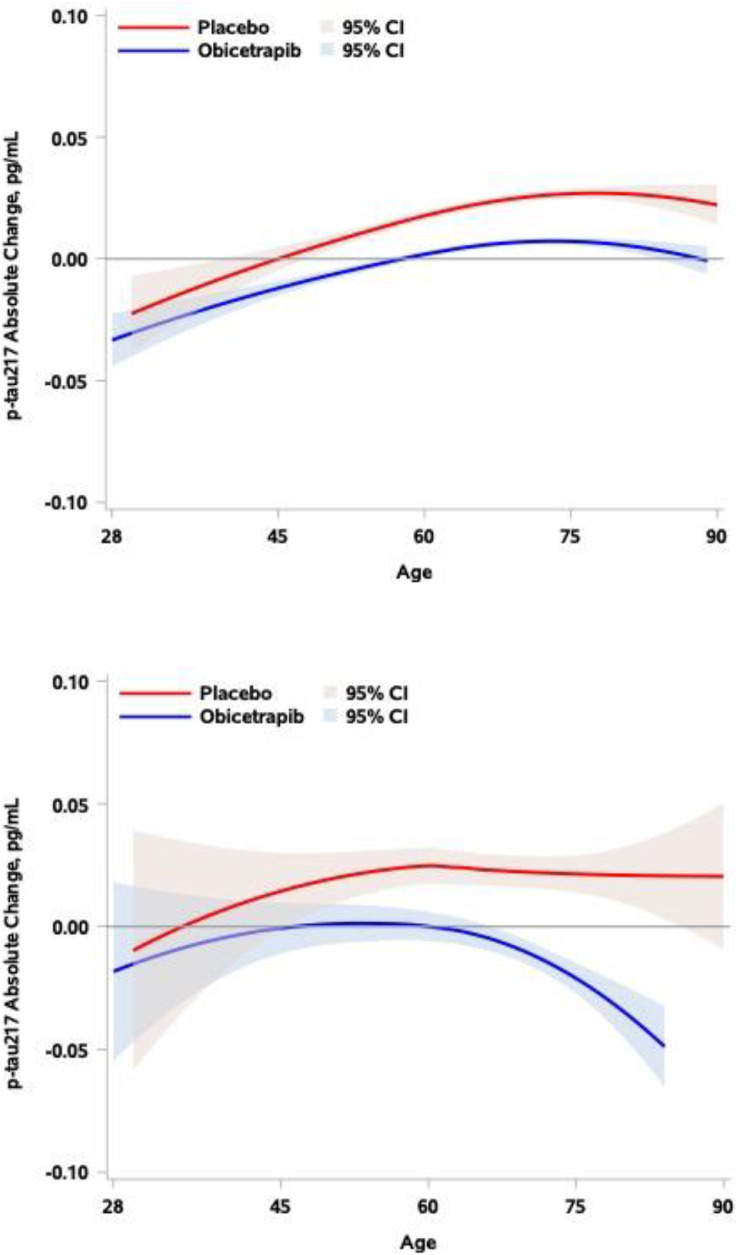
**Absolute Change in p-tau217 by Treatment Group and Continuous Age. Panel A: All Participants. Panel B: E4 Carriers.** Loess curves and corresponding 95 % CIs are degree 2 with cubic interpolation and maximum smoothing and reflect predicted values from robust regression models with terms for mean-centered baseline ptau-217, treatment group, mean-centered age, and the interaction between treatment group and age. In Panel A, the relationship between age and absolute change in p-tau217 is significant in both treatment groups (*P* = 0.0286 and *P* = 0.0001 for the placebo and obicetrapib groups, respectively), and the lack of interaction between age and treatment group (P_interaction_=0.61) indicates that there is no statistical evidence that the reduction in absolute change by obicetrapib depended on age. In Panel B, the relationship between age and absolute change in p-tau217 is not significant in either treatment group (*P* = 0.62 and *P* = 0.17 for the placebo and obicetrapib groups, respectively) and the lack of interaction between age and treatment group (P_interaction_=0.58) indicates that there is no statistical evidence that the reduction in absolute change by obicetrapib depended on age.

Fig. 3 illustrates the relationship between continuous age and absolute change in p-tau217 by treatment group for the overall analysis population (Panel A) and the subset of ApoE4 carriers (Panel B). In the placebo group, absolute increases tended to be greater among older participants in the overall analysis population (*P* = 0.029), but not in the subset of ApoE4 carriers (*P* = 0.62). The interaction p-value between age and treatment was 0.61 for the overall analysis population and 0.58 in the subset of ApoE4 carriers, indicating that the reduction in absolute change by obicetrapib relative to placebo did not depend on age.

**eFigure 1** illustrates the relationship between continuous baseline p-tau217 and absolute change in p-tau217 by treatment group for the overall analysis population (Panel A) and the subset of ApoE4 carriers (Panel B). The interaction p-value between baseline p-tau217 and treatment was <0.0001 for both the overall analysis population and the subset of ApoE4 carriers, indicating higher baseline concentrations were associated with greater decreases in p-tau217 with obicetrapib treatment relative to placebo. Furthermore, among those with baseline p-tau217 concentrations below 0.42 pg/mL, fewer participants in the obicetrapib group exceeded this threshold at study end compared to placebo [15.9 % vs 20.9 %; OR (95 % CI) = 0.54 (0.36, 0.82), *p* = 0.0041].

### 3.5 Secondary biomarker outcomes

**Aβ42:40 Ratio:** While no significant differences were demonstrated in Aβ42:40 ratios in the overall population, obicetrapib limited decreases among ApoE4/E4 participants: adjusted mean (95 % CI) = −0.36 % (−3.90 to 3.18) for obicetrapib versus −8.32 % (−13.43 to −3.21) with placebo (*p* = 0.013) (**eTable 3**).

**P-tau217/(Aβ42:40) Ratio:** Obicetrapib significantly limited increases in this composite ratio across all participants [adjusted mean (95 % CI) 2.61 % (1.00 to 4.22] versus 6.34 % (4.10 to 8.59) with placebo), representing a 4.04 % difference (*p* = 0.008). The effect was particularly pronounced in the combined E3/E4 or E4/E4 subgroups showing a 3.46 % (0.04 to 6.88) increase with obicetrapib versus 10.75 % (6.05 to 15.45) with placebo (*p* = 0.013), yielding an 7.29 % difference. ApoE4/E4 participants demonstrated a 1.67 % (−14.97 to 11.62) decrease with obicetrapib while placebo achieved a 20.98 % (5.26 to 36.69) increase, representing a 22.65 % difference in means (*p* = 0.032) (**eTable 4**).

**GFAP:** While demonstrating a trend toward benefit in the overall population, GFAP changes did not achieve statistical significance (adjusted mean (95 % CI) 1.44 % (0.25 to 2.63) for obicetrapib versus 3.40 % (1.73 to 5.07) for placebo; *p* = 0.07). However, ApoE4/E4 participants showed a significant 6.39 % (−12.78 to −0.01) decrease with obicetrapib compared to an 8.85 % (−0.58 to 17.12) increase with placebo, representing a 15.24 % difference (*p* = 0.006) (**eTable 5**).

**NfL:** Treatment effects on NfL were generally modest across most groups. ApoE4/E4 participants demonstrated a significant difference, with obicetrapib associated with an adjusted mean (95 % CI) 10.49 % (−20.84 to −0.14) decrease versus a 6.82 % (−6.12 to 19.76) increase with placebo, representing a 17.31 % difference (*p* = 0.020) (**eTable 6).**

**P-tau181:** Overall changes in p-tau181 were not significantly different between treatment groups (adjusted mean (95 % CI) 1.21 % (−0.27 to 2.68) for obicetrapib versus 1.77 % (−0.31 to 3.85) for placebo; *p* = 0.66). ApoE4/E4 participants showed a 10.51 % (−18.82 to −2.19) decrease with obicetrapib compared to a 3.16 % (−7.64 to 13.97) increase with placebo, representing a 13.67 % difference (*p* = 0.06) (**eTable 7**).

### 3.6 Summary of treatment effects and mechanistic correlations among ApoE4/E4 participants

**Fig. 4:**
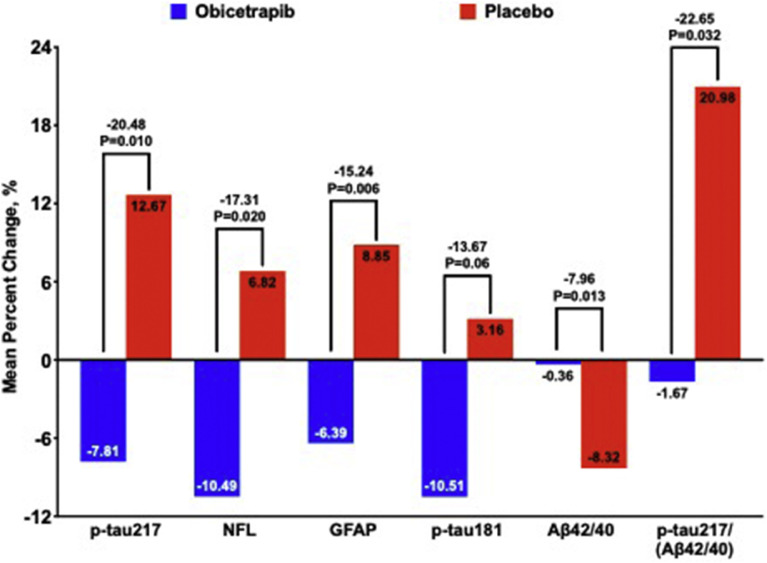
**Percent Change in AD Biomarkers Among E/E4 Participants.** Mean percent changes for each treatment group are from robust regression models with terms for treatment group, mean-centered baseline values of the biomarker, and mean-centered age.

Fig. 4 summarizes treatment effects on AD biomarkers among ApoE4/E4 participants. These findings generally suggest that the protective effects of obicetrapib were most pronounced in this subgroup. Specifically, ApoE4/E4 participants showed consistent improvements across multiple AD biomarkers compared to placebo treatment, with placebo-adjusted benefits ranging from 13.67 % to 22.65 % across different biomarkers, culminating in the placebo-adjusted 20.48 % improvement observed in p-tau217.

Correlations between lipid parameters, obicetrapib concentrations, and p-tau217 changes were examined in ApoE4/E4 participants (**eTable 8**). Higher achieved HDL-C concentrations and lower achieved LDL-C, lipoprotein(a), and apolipoprotein B levels were associated with decreases in p-tau217. Most notably, obicetrapib plasma concentrations demonstrated the strongest relationship with p-tau217 efficacy. Specifically, obicetrapib plasma concentration showed strong and statistically significant inverse correlations with p-tau217 change (*r*=−0.64 and −0.61 for absolute and percent change, respectively), indicating that higher end-of-study drug levels were associated with greater decreases in p-tau217.

Safety in the AD biomarker analysis population was not evaluated independently from the overall BROADWAY study population, where obicetrapib was observed to be well-tolerated, with a safety profile comparable to placebo.

## 4 Discussion

Obicetrapib, an oral CETP inhibitor, significantly slowed AD biomarker progression over 12 months in participants with ASCVD, with the greatest effects in ApoE4 carriers. Among ApoE4/E4 participants, obicetrapib reduced p-tau217 levels by a placebo-adjusted 20.48 %. Overall, we observed consistent effects across multiple AD biomarkers, supporting the hypothesis that obicetrapib's lipid metabolism effects may influence AD pathogenesis.

This is the first demonstration of an oral intervention that effects markers of beta-amyloid, astroglial activation, and neurodegeneration biomarkers in individuals at elevated AD risk based on age, cardiovascular factors, and APOE status. We found interactions between baseline p-tau217 concentrations and treatment response across all participants, with ApoE4 carriers showing larger treatment effects. Higher baseline biomarkers concentrations were associated with greater increases with placebo but correspondingly greater decreases with obicetrapib treatment. Fewer participants receiving obicetrapib exceeded the pathological threshold of 0.42 pg/mL for p-tau217 at the end of study, suggesting potential for preventing or delaying the transition to elevated AD biomarker status.

### 4.1 Clinical context and current treatment gaps

APOE4 carriers face elevated AD risk but have limited prevention options at this time. Existing prevention strategies center on lifestyle modifications: cognitive training, physical exercise, Mediterranean-style diets, and cardiovascular risk management. The FINGER trial showed that multidomain lifestyle interventions can slow cognitive decline in at-risk older adults by 25 % over two years [29,30]. These results were replicated in the US Pointer study [31].

Prior randomized trials of statins, including simvastatin in patients with mild to moderate AD (CLASP-AD trial) and atorvastatin for prevention in mild to moderate probable AD (LEADe study), failed to demonstrate cognitive benefits [32,33]. Several factors may explain why our observations with obicetrapib differ from the statin trials. Statins primarily reduce LDL-C without affecting other atherogenic lipoproteins or raise HDL-C. Obicetrapib not only lowers LDL-C and other atherogenic lipoproteins, but also significantly increases functional HDL-C and improves HDL particle functionality, which could provide neuroprotection through antioxidant and anti-inflammatory effects. The statin trials enrolled patients with established dementia, where neurodegeneration was likely too advanced to reverse, whereas the BROADWAY AD substudy examined individuals with cardiovascular disease who were asymptomatic and cognitively intact at baseline, representing an earlier stage in the AD continuum when biomarker changes might still be modifiable. CETP is expressed in brain tissue, particularly astrocytes, where it directly influences CNS cholesterol balance, a mechanism distinct from peripheral LDL-lowering achieved by statins. Moreover, genetic studies have linked lower CETP activity with preserved cognition and reduced dementia risk in population analyses, whereas similar protective genetic associations have not been established for 3‑hydroxy-3-methylglutaryl-CoA reductase (HMGCR) pathways [34]. These differences suggest CETP inhibition may work through fundamentally different mechanisms than conventional statin therapy for Alzheimer’s prevention.

Anti-amyloid monoclonal antibodies have provided new treatment options for symptomatic AD. However, prevention approaches for asymptomatic individuals with genetic risk factors remain under investigation. For APOE4 carriers, oral therapies that affect both amyloid and tau biomarkers could address current gaps in prevention options [[35], [36], [37]].

The relationship between cardiovascular risk factors and AD pathology has led to investigation of cardiometabolic approaches in prevention research [38,39]. Studies indicate that vascular health and metabolic health are both positively associated with cognitive outcomes during aging. This has expanded prevention research to include interventions that may influence both cardiovascular and neurological pathways. Such approaches may be relevant for individuals with combined cardiovascular and genetic risk factors. While clinical outcomes data are eagerly awaited, this area of research provides additional avenues for exploring prevention strategies in at-risk populations [[40], [41], [42]].

### 4.2 Understanding the mechanisms

Obicetrapib represents a mechanistically distinct approach to AD prevention. Rather than targeting downstream pathology like anti-amyloid therapies, obicetrapib addresses upstream lipid dysregulation, oxidation, and inflammation that may drive disease pathogenesis, especially in APOE4 carriers, as suggested by genetic studies.

Obicetrapib's effect on lipids directly impact cerebrovascular health through antiatherogenic benefits. By reducing LDL-C, increasing HDL-C, and decreasing lipoprotein(a), obicetrapib provides a lipid environment that may enhance blood-brain barrier (BBB) integrity [43]. HDL promotes cerebrovascular health by reducing arterial plaque buildup, ensuring adequate oxygen and nutrient delivery to the brain. This potentially lowers risks of both vascular dementia and AD-related brain changes. Improved vascular health is particularly relevant for APOE4 carriers, who exhibit greater atherosclerosis, microvascular damage, and compromise of BBB integrity, making them especially susceptible to vascular contributions to cognitive impairment [44,45].

Small functional HDL particles increased by obicetrapib might provide neuroprotection. HDL may directly facilitate amyloid-beta clearance by binding to these proteins, reducing their toxicity and aiding removal from the brain [46]. Higher HDL levels are associated with better cognitive function and lower AD risk in older adults. Meta-analyses show that higher HDL levels correlated with 10–15 % reduced AD risk [47].

Obicetrapib increases levels of lipophilic antioxidants including lutein, zeaxanthin, and alpha-tocopherol in both HDL and cerebrospinal fluid [48,49]. These carotenoids and vitamin E compounds provide protection against lipid peroxidation and oxygen radical formation, which are stimuli for neuroinflammation. Chronic inflammation contributes to AD pathogenesis, making HDL's anti-inflammatory effects particularly relevant. Higher HDL particle levels have been linked to lower brain inflammation markers, potentially reducing neuroinflammation and protecting neurons [50]. This aligns with our observed effects on GFAP, a marker of astroglial activation.

HDL transports key omega-3 fatty acids such as DHA to the brain, where carotenoids carried by HDL particles protect these polyunsaturated fatty acids (PUFA) from oxidation. By increasing small functional HDL particles and elevating brain carotenoid levels, obicetrapib may provide protection for brain PUFAs including DHA [51,52].

Small functional HDL particles are also relevant for brain cholesterol metabolism where HDL-like particles involving ApoE protein regulate cholesterol transport [53]. Since APOE4 represents a major AD risk factor, obicetrapib's ability to increase HDL functionality may improve cholesterol homeostasis and reduce APOE4-related risk. Small HDL particles, increased by 30 % with obicetrapib, can cross the BBB endothelium through holoparticle uptake mechanisms [54,55]. Both HDL particles and apoA1 inhibit amyloid-beta aggregation. Research has shown that peripheral overexpression of human apoA1 preserves cognitive function, reduces neuroinflammation, and protects against cerebral amyloid angiopathy in mice, suggesting a direct role for peripheral small functional HDL particles in brain amyloid-beta clearance [56,57].

### 4.3 Study considerations

Several limitations must be acknowledged when interpreting these results. Although ApoE phenotyping was unknown for 35 % of participants, and 4 % were excluded for other reasons, baseline characteristics were generally similar between our analysis population and those who were excluded, with differences primarily limited to lipids/lipoproteins. These similarities suggest that the excluded participants did not compromise the external validity of our findings. The small number of ApoE4 homozygotes limits effect estimate precision in this subgroup despite observed statistical significance. Given the design of the BROADWAY trial, we did not assess cognitive function or clinical outcomes in this analysis, focusing exclusively on biomarker endpoints. The clinical significance of these biomarker changes will be interrogated through studies including cognitive outcomes. The 12-month duration, adequate for detecting biomarker changes, may not fully capture long-term biomarker trajectory. Our observed mean Aβ42:40 ratio of 0.058 is consistent with recently published values from similar cardiovascular populations (0.061), indicating our measurements fall within expected ranges [22].

Given obicetrapib's effects on lipid metabolism and lipoprotein composition, we considered whether obicetrapib might interfere with the p-tau217 assay. Several factors argue against assay interference: Obicetrapib’s effects differed across APOE subgroups, with the strongest impact seen in E4/E4 carriers, the group at the highest AD risk with the most pronounced lipid abnormalities. We also observed consistent effects across multiple biomarkers (p-tau217, p-tau181, GFAP, NfL, Aβ42:40) that were measured using different assay platforms, making it unlikely that assay interference explains all findings. The correlation between obicetrapib plasma levels and biomarker changes points to a biological effect rather than a measurement artifact. Finally, validation studies have shown the SIMOA p-tau217 assay tolerated lipid samples without interference [58].

The strong correlation between obicetrapib plasma concentrations and p-tau217 changes (*r*=−0.64, *P* = 0.0002) supports CETP inhibition as the underlying mechanism, though correlation alone does not establish causality. While the dose-response pattern and the biological plausibility of CETP-mediated effects are consistent with this interpretation, we have not fully characterized the pathways involved or ruled out other contributing mechanisms. Future studies that directly measure CETP activity and lipoprotein particle characteristics alongside AD biomarkers will help clarify how CETP inhibition produced these effects.

Beyond understanding the mechanism, questions remain about clinical significance. Plasma p-tau217 correlates well with brain amyloid and tau pathology on PET scans and in CSF samples from cross-sectional studies [59]. But whether lowering plasma p-tau217 with treatment slows cognitive decline or prevents AD has not been established. The clinical significance of what we observed here can only be established through prospective trials that include cognitive testing, brain imaging, and CSF biomarker measurements.

Our findings may have implications for AD prevention, particularly for APOE4 patients who currently have no effective prevention options. The ability to reduce pathological biomarker progression suggests potential for altering disease trajectory in this population. The established safety profile of obicetrapib, demonstrated across multiple large clinical trials, including older individuals, combined with its oral administration, may facilitate clinical implementation, when approved.

The therapeutic potential of obicetrapib gains additional relevance given recent findings demonstrating the burden of undiagnosed cognitive impairment among patients with cardiovascular disease. A recent prospective study of individuals with cardiovascular risk factors and established ASCVD reported that 29 % had cognitive performance consistent with MCI on standardized testing, despite lacking formal diagnoses. Moreover, the observation that 55 % of these participants have elevated p-tau217 levels underscores the widespread nature of AD pathology in cardiovascular populations [22]. Our demonstration that obicetrapib attenuates p-tau217 progression in the BROADWAY population suggests potential utility not only for primary prevention but also for the proportion of cardiovascular patients who may already have early-stage cognitive impairment that remains clinically unrecognized.

## 5 Conclusions

This study demonstrates that obicetrapib is associated with effects on AD biomarkers, especially in E4 carriers. Reducing AD pathology progression in this high-risk population could represent an advance in disease prevention. The mechanistic rationale, supported by genetic evidence and biomarker effects, suggests that targeting lipid metabolism through CETP inhibition with obicetrapib offers a novel approach to AD prevention. Further research is needed to confirm cognitive benefits. Our findings provide evidence for a new direction in AD prevention research.

## Funding

Funded by NewAmsterdam Pharma; BROADWAY ClinicalTrials.gov number, NCT05142722

## Disclosures

**Stephen J Nicholls:** received grant/research support from AstraZeneca, NewAmsterdam Pharma, Amgen, Anthera, Cyclarity, Eli Lilly, Esperion, Novartis, Cerenis, The Medicines Company, Resverlogix, InfraReDx, Roche, Sanofi-Regeneron, and LipoScience; and was a consultant for Abcentra, AstraZeneca, Amarin, Akcea, Eli Lilly, Anthera, Omthera, Merck, Takeda, Resverlogix, Sanofi-Regeneron, CSL Behring, Esperion, Boehringer Ingelheim, Daiichi Sankyo, Silence Therapeutics, CSL Seqirus and Vaxxinity.

**Kausik K Ray:** reports Research grants- Amgen, Sanofi, Regeneron, Daiichi Sankyo, and Ultragenyx to Imperial College London. Consultancy-Novartis, Daiichi Sankyo, Kowa, Esperion, Novo Nordisk, MSD, Lilly, Silence Therapeutics, AZ, NewAmsterdam Pharma, Bayer, Beren Therapeutics, Cleerly, EmendoBio, Scribe, Crispr, Vaxxinity, Amarin, Regeneron, Ultragenyx, Sanofi Cargene, and Resverlogix. Fees for lectures-Novartis, BI, AZ, Novo Nordisk, Viatris, Amarin, Biologix Pharma, Sanofi, Amgen, Esperion, Daiichi Sankyo, Mankind, Macleod Pharma for symposia at international meetings. Holding stock options from NewAmsterdam Pharma, SCRIBE therapeutics and Pemi31.

**Michael Szarek:** reports receiving salary support from CPC, a non-profit academic research organization affiliated with the University of Colorado, that receives or has received research grant/consulting funding between July 2021 and July 2024 from the following organizations: Abbott Laboratories, Agios Pharmaceuticals, Inc., Alexion Pharma Godo Kaisha, Amgen Inc., Anthos Therapeutics, Inc., ARCA biopharma, Inc., Arrowhead Pharmaceuticals, AstraZeneca Pharma India, AstraZeneca UK Ltd, Bayer, Bayer Aktiengesellschaft, Bayer Pharma AG, Beth Israel Deaconess Medical Center, Better Therapeutics, Boston Clinical Research Institute, LLC, Bristol-Myers Squibb, Cleerly, Inc., Colorado Dept of Public Health and Environment, Congress Inc, Cook Regentec LLC, CSL Behring LLC, Eidos Therapeutics, Inc., EPG Communication Holdings Ltd., Esperion Therapeutics, Inc, Faraday Pharmaceuticals, Inc., HeartFlow Inc, Insmed, Ionis Pharmaceuticals, IQVIA Inc., Janssen Pharmaceuticals, Inc, Janssen Research & Development, LLC, Janssen Scientific Affairs LLC, Lexicon Pharmaceuticals, Inc., Medpace, Inc., Medscape, Merck Sharp & Dohme Corp., Nectero Medical, Inc, Novartis Pharmaceuticals Corporation, Novo Nordisk Inc., Pfizer, PPD Development, L.P., Prothena Biosciences Limited, Regeneron, Regents of the University of Colorado (aka UCD), Sanifit Therapeutics S.A., Sanofi, Silence Therapeutics PLC, Stanford University, Stealth BioTherapeutics Inc., The Brigham and Women's Hospital, Thrombosis Research Institute, Tourmaline Bio, Inc, University of Colorado, University of Colorado Denver, University of Pittsburgh, VarmX, Verve Therapeutics, WraSer, LLC. Dr. Szarek also reports serving as a consultant or research support (or both) from Amarin, Lexicon, NewAmsterdam, Novartis, Regeneron, Sanofi, Silence, and Tourmaline.

**Everard Vijverberg:** received consultancy fees (paid to the university) for New Amsterdam Pharma, Treeway, ReMynd, Vivoryon, Biogen, Vigil Neuroscience, ImmunoBrain Checkpoint, Muna Therapeutics, Esai, Eli Lilly, CogRX, Therini, UCB and Roche. Within his university affiliation he is PI of studies of DIAN, AC immune, Alnylam, CogRX therapeutics, New Amsterdam Pharma, Janssen, UCB, Roche, Vivoryon, ImmunoBrain, GSK, MSD, Biogen, Alector, Eli Lilly, AriBio Fuij Film Toyama, GemVax. Co-founder van het CANDIDATE Center Amsterdam UMC. Scientific projects with the Dutch Soccer Association (KNVB)

**Philip Scheltens:** is a full time employee of EQT LifeSciences, emeritus prof at Amsterdam University Medical Center, and consultant to New Amsterdam Pharma

**Jeffrey L. Cummings:** has provided consultation to Acadia, Acumen, ALZpath, AnnovisBio, Artery, Axsome, Biogen, Biohaven, Bristol-Myers Squib, Eisai, Fosun, GAP Foundation, Hummingbird Diagnostics. IGC, Janssen, Julius Clinical, Kinoxis, Lighthouse, Lilly, Lundbeck, LSP/eqt, Merck, MoCA Cognition, Novo Nordisk, NSC Therapeutics, Optoceutics, Otsuka, Praxis, ReMYND, Roche, Scottish Brain Sciences, Signant Health, Simcere, sinaptica, T-Neuro, TrueBinding, and Vaxxinity pharmaceutical, assessment, and investment companies. Dr. Cummings is co-founder of CNS Innovations and Mangrove Therapeutics. Dr. Cummings is supported by NIGMS grant P20GM109025; NIA R35AG71476; NINDS RO1NS139383; Alzheimer’s Disease Drug Discovery Foundation (ADDF); Ted and Maria Quirk Endowment; Joy Chambers-Grundy Endowment.

**Michael H Davidson, John JP Kastelein, Danielle L Curcio, Douglas Kling, Marc Ditmarsch, and Andrew Hsieh** are employees of NewAmsterdam Pharma and hold stocks or options.

## CRediT authorship contribution statement

**Michael H Davidson:** Writing – review & editing, Supervision, Funding acquisition, Data curation, Conceptualization. **Michael Szarek:** Writing – review & editing, Writing – original draft, Formal analysis, Data curation, Conceptualization. **Philip Scheltens:** Writing – review & editing, Investigation, Data curation, Conceptualization. **Everard Vijverberg:** Writing – review & editing, Data curation, Conceptualization. **Andrew Hsieh:** Writing – review & editing, Writing – original draft, Data curation, Conceptualization. **Marc Ditmarsch:** Writing – review & editing, Data curation, Conceptualization. **Douglas Kling:** Writing – review & editing, Project administration, Data curation, Conceptualization. **Danielle Curcio:** Writing – review & editing, Data curation, Conceptualization. **Stephen J Nicholls:** Writing – review & editing, Writing – original draft, Methodology, Formal analysis, Data curation, Conceptualization. **Kausik K Ray:** Writing – review & editing, Data curation, Conceptualization. **Jeffrey L. Cummings:** Writing – review & editing, Supervision, Conceptualization. **John JP Kastelein:** Writing – review & editing, Writing – original draft, Methodology, Funding acquisition, Data curation, Conceptualization.

## Declaration of competing interest

The authors declare the following financial interests/personal relationships which may be considered as potential competing interests: Scheltens P reports statistical analysis was provided by Amsterdam UMC Location VUmc. If there are other authors, they declare that they have no known competing financial interests or personal relationships that could have appeared to influence the work reported in this paper.

## Appendix Supplementary materials

Image, application 1
