# Supplementary material for: Effect of obicetrapib, a potent cholesteryl ester transfer protein inhibitor, on p-tau217 levels in patients with cardiovascular disease
Source: J Prev Alzheimers Dis. 2025 Oct 17;13(1):100394. doi: 10.1016/j.tjpad.2025.100394 (PMC12811769; doi:10.1016/j.tjpad.2025.100394)
Supplement: Supplementary file 1 [file mmc1.docx]

**eFigure 1. Absolute Change in p-tau217 by Treatment Group and Continuous Baseline p-tau217. Panel A: All Participants. Pabel B: E4 Carriers.** Loess curves and corresponding 95% CIs are degree 2 with cubic interpolation and maximum smoothing and reflect predicted values from robust regression models with terms for mean-centered baseline p-tau217, treatment group, mean-centered age, and the interaction between treatment group and baseline p-tau217. In Panel A, the relationship between baseline p-tau217 and absolute change in p-tau217 was not significant in the placebo group (P=0.95) but was significant in the obicetrapib group (P<0.0001). Coupled with the significant interaction between baseline p-tau217 and treatment group (P_interaction_<0.0001), there results indicate higher baseline concentrations were associated with greater decreases in p-tau217 with obicetrapib treatment relative to placebo. In Panel B, the relationship between baseline p-tau217 and absolute change in p-tau217 was significant in both treatment groups (P=0.0228 and P<0.0001) for the placebo and obicetrapib groups, respectively). Coupled with the significant interaction between baseline p-tau217 and treatment group (P_interaction_<0.0001) there results indicate higher baseline concentrations were associated with greater decreases in p-tau217 with obicetrapib treatment relative to placebo.

**eFigure 1. Absolute Change in p-tau217 by Treatment Group and Continuous Baseline p-tau217. Panel A: All Participants. Pabel B: E4 Carriers.**

**Panel A**

**Panel B**

**eTable 1. Additional Baseline Characteristics**

|  | **E3/E3** | | **E2/E2, E2/E3** | | **E2/E4** | | **E3/E4** | | **E4/E4** | | **P-value*** |
| --- | --- | --- | --- | --- | --- | --- | --- | --- | --- | --- | --- |
|  | **Obicetrapib**  **(n=695)** | **Placebo**  **(n=350)** | **Obicetrapib**  **(n=65)** | **Placebo**  **(n=38)** | **Obicetrapib**  **(n=13)** | **Placebo**  **(n=7)** | **Obicetrapib**  **(n=225)** | **Placebo**  **(n=113)** | **Obicetrapib**  **(n=19)** | **Placebo**  **(n=10)** |  |
| BMI, kg/m^2^ | 29.5 (25.9, 33.0) | 29.3 (26.4, 32.4) | 29.2 (26.5, 33.6) | 29.4 (25.1, 33.2) | 29.4 (26.1, 32.3) | 28.2 (26.3, 31.4) | 29.7 (26.2, 33.7) | 31.1 (26.9, 34.5) | 27.7 (24.2, 34.8) | 26.6 (25.6, 30.7) | 0.29 |
| eGFR, mL/min/1.73m^2^ | 88 (72, 97) | 86 (72, 97) | 80 (66, 96) | 88 (75, 97) | 93 (78, 99) | 96 (87, 98) | 89 (73, 97) | 88 (72, 97) | 93 (80, 96) | 92 (82, 99) | 0.16 |
| hsCRP, mg/L | 1.3 (0.6, 3.4) | 1.5 (0.8, 3.3) | 1.3 (0.7, 3.4) | 2.1 (0.8, 4.9) | 0.7 (0.6, 1.4) | 0.9 (0.5, 4.1) | 1.0 (0.5, 2.8) | 1.0 (0.5, 2.4) | 1.0 (0.3, 3.0) | 1.2 (0.4, 3.3) | 0.0014 |
| HbA1c, % | 5.9 (5.6, 6.6) | 6.0 (5.6, 6.7) | 5.8 (5.6, 6.3) | 5.9 (5.6, 6.4) | 5.7 (5.7, 6.6) | 5.9 (5.4, 7.5) | 5.9 (5.6, 6.5) | 6.0 (5.6, 6.5) | 5.9 (5.7, 6.1) | 6.2 (5.7, 6.8) | 0.80 |
| Fasting Blood Glucose, mg/dL | 106 (97, 123) | 109 (98, 128) | 105 (97, 120) | 102 (93, 121) | 101 (96, 118) | 108 (92, 158) | 105 (96, 123) | 108 (99, 126) | 106 (101, 122) | 111 (97, 120) | 0.58 |
| Randomization Strata  HeFH  High Intensity Statin | 134 (19.3)  570 (82.0) | 74 (21.1)  290 (82.9) | 12 (18.5)  52 (80.0) | 8 (21.1)  32 (84.2) | 3 (23.1)  11 (84.6) | 2 (28.6)  6 (85.7) | 41 (18.2)  184 (81.8) | 18 (15.9)  102 (90.3) | 6 (31.6)  14 (73.7) | 0  8 (80.0) | 0.84  0.72 |
| Lipids/Lipoproteins  LDL-C (M-H), mg/dL  HDL-C, mg/dL  Lp(a), nmol/L  ApoB, mg/dL  ApoA1, mg/dL  Non-HDL-C, mg/dL  Triglyceride, mg/dL | 86 (71, 113)  49 (40, 58)  36.0 (9.7, 156.8)  84 (71, 101)  155 (137, 176)  110 (92, 139)  117 (89, 164) | 89 (72, 117)  48 (40, 58)  36.1 (11.3, 150.2)  86 (73, 104)  155 (141, 175)  112 (92, 142)  129 (91, 181) | 82 (64, 105)  46 (38, 58)  36.5 (10.2, 185.5)  78 (67, 93)  160 (137, 175)  108 (88, 134)  148 (111, 191) | 80 (69, 113)  52 (37, 63)  15.4 (4.8, 85.3)  77 (61, 92)  158 (139, 175)  109 (88, 135)  116 (82, 195) | 87 (75, 110)  51 (33, 62)  45.5 (7.4, 261.5)  93 (79, 111)  162 (135, 175)  118 (97, 155)  128 (78, 215) | 69 (64, 84)  45 (36, 56)  123.9 (3.9, 263.4)  71 (68, 82)  150 (139, 168)  100 (91, 115)  128 (98, 182) | 87 (72, 111)  49 (40, 61)  40.8 (8.6, 185.9)  83 (72, 101)  155 (138, 172)  110 (92, 137)  117 (86, 174) | 86 (70, 110)  49 (40, 59)  65.1 (14.5, 209.7)  83 (70, 102)  154 (138, 172)  107 (89, 136)  121 (90, 174) | 90 (73, 100)  53 (43, 61)  124.8 (27.6, 214.9)  83 (74, 99)  157 (137, 180)  107 (90, 127)  99 (85, 110) | 84 (73, 112)  43 (38, 53)  12.1 (4.2, 32.5)  94 (73, 108)  146 (135, 155)  114 (87, 151)  119 (99, 175) | 0.0027  0.88  0.14  0.0032  0.88  0.65  0.10 |

Note: values in table are n (%) or median (Q1, Q3)

*P-values for comparisons between ApoE status (pooled across treatment groups) by Kruskal-Wallis or chi-square tests

**eTable 2. Baseline Characteristics for BROADWAY Participants Included Versus Excluded from the Analysis Population**

|  | **Included**  **(n=1535)** | **Excluded**  **(n=995)** |
| --- | --- | --- |
| Age, years | 67 (61, 73) | 65 (59, 71) |
| Female sex | 506 (33.0) | 347 (34.9) |
| Race  White  Asian  Black  Other | 1297 (84.5)  133 (8.7)  86 (5.6)  19 (1.2) | 591 (59.4)  329 (33.1)  65 (6.5)  10 (1.0) |
| Medical History  Diabetes  Hypertension  ASCVD | 580 (37.8)  1301 (84.8)  1337 (87.1) | 380 (38.2)  730 (73.4)  914 (91.9) |
| BMI, kg/m^2^ | 29.5 (26.1, 33.1) | 27.8 (25.0, 31.5) |
| eGFR, mL/min/1.73m^2^ | 88 (72, 97) | 89 (73, 99) |
| hsCRP, mg/L | 1.3 (0.6, 3.3) | 1.4 (0.6, 3.4) |
| HbA1c, % | 5.9 (5.6, 6.6) | 5.9 (5.5, 6.6) |
| Fasting Blood Glucose, mg/dL | 106 (97, 125) | 108 (97, 129) |
| Randomization Strata  HeFH  High Intensity Statin | 298 (19.4)  1269 (82.7) | 129 (13.0)  505 (50.8) |
| Lipids/Lipoproteins  LDL-C (M-H), mg/dL  HDL-C, mg/dL  Lp(a), nmol/L  ApoB, mg/dL  ApoA1, mg/dL  Non-HDL-C, mg/dL  Triglyceride, mg/dL | 87 (71, 113)  49 (40, 59)  37.1 (10.1, 163.8)  83 (71, 102)  155 (138, 175)  110 (91, 140)  122 (89, 174) | 98 (80, 127)  46 (38, 56)  44.4 (13.1, 164.0)  91 (77, 109)  150 (134, 166)  123 (102, 155)  127 (92, 171) |

Note: values in table are n (%) or median (Q1, Q3) and [*number of observations*]. ASCVD = atherosclerotic cardiovascular disease

**eTable 3. Change in AB42/40 from Baseline to End of Study**

|  | **Unadjusted** | | | **Adjusted*** | | |
| --- | --- | --- | --- | --- | --- | --- |
|  | **Obicetrapib** | **Placebo** | **P-value** | **Obicetrapib** | **Placebo** | **P-value** |
| All Participants |  |  |  |  |  |  |
| Absolute Change, pg/mL | -0.0002 (-0.0005, 0.0002) | -0.0005 (-0.0010, 0) | 0.25 | -0.0003 (-0.0007, 0) | -0.0007 (-0.0012, -0.0002) | 0.21 |
| Percent Change, % | -0.40 (-1.03, 0.23) | -1.07 (-1.95, -0.19) | 0.23 | -0.44 (-1.06, 0.19) | -1.09 (-1.97, -0.21) | 0.23 |
| E3/E4, E4/E4 |  |  |  |  |  |  |
| Absolute Change, pg/mL | -0.0005 (-0.0012, 0.0003) | -0.0014 (-0.0024, -0.0003) | 0.18 | -0.0010 (-0.0018, -0.0003) | -0.0023 (-0.0033, -0.0013) | 0.05 |
| Percent Change, % | -1.03 (-2.43, 0.38) | -2.84 (-4.82, -0.86) | 0.14 | -1.74 (-3.13, -0.35) | -3.77 (-5.72, -1.82) | 0.10 |
| E3/E4 |  |  |  |  |  |  |
| Absolute Change, pg/mL | -0.0005 (-0.0013, 0.0003) | -0.0011 (-0.0022, 0.0001) | 0.41 | -0.0012 (-0.0019, -0.0004) | -0.0021 (-0.0033, -0.0010) | 0.16 |
| Percent Change, % | -1.09 (-2.61, 0.43) | -2.21 (-4.34, -0.07) | 0.40 | -1.79 (-3.28, -0.30) | -3.18 (-5.26, -1.10) | 0.28 |
| E4/E4 |  |  |  |  |  |  |
| Absolute Change, pg/mL | -0.0006 (-0.0025, 0.0013) | -0.0041 (-0.0069, -0.0013) | 0.0387 | -0.0008 (-0.0025, 0.0009) | -0.0045 (-0.0070, -0.0020) | 0.0188 |
| Percent Change, % | -0.37 (-3.81, 3.06) | -8.02 (-13.01, -3.02) | 0.0135 | -0.36 (-3.90, 3.18) | -8.32 (-13.43, -3.21) | 0.0126 |

Note: values in table are mean (95% CI)

*Results reflect adjustment for mean-centered baseline AB42/40 and mean-centered age.

**eTable 4. Change in p-tau217/(AB42/40) from Baseline to End of Study**

|  | **Unadjusted** | | | **Adjusted*** | | |
| --- | --- | --- | --- | --- | --- | --- |
|  | **Obicetrapib** | **Placebo** | **P-value** | **Obicetrapib** | **Placebo** | **P-value** |
| All Participants |  |  |  |  |  |  |
| Absolute Change, pg/mL | 0.167 (0.037, 0.298) | 0.543 (0.360, 0.725) | 0.0010 | 0.136 (0.006, 0.267) | 0.50 (0.319, 0.684) | 0.0014 |
| Percent Change, % | 2.57 (0.96, 4.19) | 6.50 (4.25, 8.76) | 0.0018 | 2.61 (1.00, 4.22) | 6.34 (4.10, 8.59) | 0.0080 |
| E3/E4, E4/E4 |  |  |  |  |  |  |
| Absolute Change, pg/mL | 0.115 (-0.196, 0.425) | 0.941 (0.505, 1.378) | 0.0025 | 0.125 (-0.196, 0.447) | 0.926 (0.485, 1.366) | 0.0036 |
| Percent Change, % | 2.28 (-0.89, 5.44) | 11.25 (6.80, 15.70) | 0.0013 | 2.95 (-0.32, 6.23) | 11.61 (7.13, 16.10) | 0.0020 |
| E3/E4 |  |  |  |  |  |  |
| Absolute Change, pg/mL | 0.149 (-0.161, 0.460) | 0.869 (0.433, 1.304) | 0.0083 | 0.159 (-0.164, 0.480) | 0.856 (0.415, 1.298) | 0.0112 |
| Percent Change, % | 2.90 (-0.41, 6.22) | 10.45 (5.80, 15.09) | 0.0096 | 3.46 (0.04, 6.88) | 10.75 (6.05, 15.45) | 0.0127 |
| E4/E4 |  |  |  |  |  |  |
| Absolute Change, pg/mL | -0.767 (-2,214, 0.680) | 2.305 (0.202, 4.408) | 0.0183 | -0.461 (-2.363, 1.442) | 2.255 (0.006, 4.504) | 0.07 |
| Percent Change, % | -4.19 (-14.59, 6.21) | 20.91 (5.79, 36.03) | 0.0073 | -1.67 (-14.97, 11.62) | 20.98 (5.26, 36.69) | 0.0324 |

Note: values in table are mean (95% CI)

*Results reflect adjustment for mean-centered baseline p-tau217/(AB42/40) and mean-centered age

**eTable 5. Change in GFAP from Baseline to End of Study**

|  | **Unadjusted** | | | **Adjusted*** | | |
| --- | --- | --- | --- | --- | --- | --- |
|  | **Obicetrapib** | **Placebo** | **P-value** | **Obicetrapib** | **Placebo** | **P-value** |
| All Participants |  |  |  |  |  |  |
| Absolute Change, pg/mL | 1.51 (0.35, 2.67) | 2.86 (1.24, 4.48) | 0.18 | 1.17 (0.01, 2.32) | 2.01 (0.40, 3.62) | 0.42 |
| Percent Change, % | 1.20 (0.01, 2.40) | 3.68 (2.01, 5.35) | 0.0194 | 1.44 (0.25, 2.63) | 3.40 (1.73, 5.07) | 0.07 |
| E3/E4, E4/E4 |  |  |  |  |  |  |
| Absolute Change, pg/mL | 0.99 (-1.52, 3.49) | 2.81 (-0.69, 6.31) | 0.41 | 1.28 (-1.29, 3.85) | 1.95 (-1.57, 5.46) | 0.76 |
| Percent Change, % | 0.43 (-1.90, 2.77) | 3.21 (-0.05, 6.48) | 0.17 | 1.51 (-0.87, 3.89) | 3.02 (-0.24, 6.28) | 0.46 |
| E3/E4 |  |  |  |  |  |  |
| Absolute Change, pg/mL | 1.78 (-0.90, 4.46) | 2.18 (-1.56, 5.93) | 0.87 | 1.97 (-0.76, 4.70) | 1.37 (-2.38, 5.12) | 0.80 |
| Percent Change, % | 1.18 (-1.33, 3.69) | 2.64 (-0.87, 6.15) | 0.51 | 2.36 (-0.21, 4.92) | 2.38 (-1.16, 5.91) | 0.99 |
| E4/E4 |  |  |  |  |  |  |
| Absolute Change, pg/mL | -5.99 (-11.90, -0.08) | 8.54 (0.39, 16.68) | 0.0047 | -7.28 (-13.90, 0.66) | 9.31 (0.74, 17.89) | 0.0040 |
| Percent Change, % | -5.88 (-11.58, -0.19) | 8.49 (0.64, 16.34) | 0.0037 | -6.39 (-12.78, -0.01) | 8.85 (-0.58, 17.12) | 0.0062 |

Note: values in table are mean (95% CI)

*Results reflect adjustment for mean-centered baseline GFAP and mean-centered age.

**eTable 6. Change in NfL from Baseline to End of Study**

|  | **Unadjusted** | | | **Adjusted*** | | |
| --- | --- | --- | --- | --- | --- | --- |
|  | **Obicetrapib** | **Placebo** | **P-value** | **Obicetrapib** | **Placebo** | **P-value** |
| All Participants |  |  |  |  |  |  |
| Absolute Change, pg/mL | 0.58 (0.25, 0.92) | 0.20 (-0.27, 0.67) | 0.19 | -0.34 (-0.67, -0.01) | -0.62 (-1.08, -0.16) | 0.32 |
| Percent Change, % | 1.94 (0.16, 3.72) | 0.69 (-1.80, 3.19) | 0.42 | -1.22 (-2.90, 0.45) | -2.08 (-4.40, 0.25) | 0.56 |
| E3/E4, E4/E4 |  |  |  |  |  |  |
| Absolute Change, pg/mL | 0.19 (-0.48, 0.87) | -0.29 (-1.23, 0.65) | 0.41 | -0.97 (-1.64, -0.30) | -1.38 (-2.29, -0.46) | 0.48 |
| Percent Change, % | -0.31 (-4.12, 3.51) | -1.92 (-7.19, 3.35) | 0.63 | -4.08 (-7.49, -0.66) | -5.04 (-9.69, -0.40) | 0.74 |
| E3/E4 |  |  |  |  |  |  |
| Absolute Change, pg/mL | 0.31 (-0.42, 1.04) | -0.45 (-1.47, 0.56) | 0.23 | -0.90 (-1.63, -0.17) | -1.54 (-2.54, -0.54) | 0.31 |
| Percent Change, % | -0.03 (-4.13, 4.08) | -2.88 (-8.60, 2.84) | 0.43 | -3.68 (-7.29, -0.07) | -5.20 (-10.16, -0.24) | 0.63 |
| E4/E4 |  |  |  |  |  |  |
| Absolute Change, pg/mL | -0.96 (-2.87, 0.95) | 1.04 (-1.38, 3.45) | 0.20 | -1.60 (-3.72, 0.52) | -0.25 (-2.90, 2.40) | 0.38 |
| Percent Change, % | -3.50 (-13.80, 6.80) | 3.82 (-9.21, 16.85) | 0.39 | -10.49 (-20.84, -0.14) | 6.82 (-6.12, 19.76) | 0.0203 |

Note: values in table are mean (95% CI)

*Results reflect adjustment for mean-centered baseline NfL and mean-centered age.

**eTable 7. Change in p-tau181 from Baseline to End of Study**

|  | **Unadjusted** | | | **Adjusted*** | | |
| --- | --- | --- | --- | --- | --- | --- |
|  | **Obicetrapib** | **Placebo** | **P-value** | **Obicetrapib** | **Placebo** | **P-value** |
| All Participants |  |  |  |  |  |  |
| Absolute Change, pg/mL | 0.29 (-0.01, 0.58) | 0.55 (0.13, 0.97) | 0.31 | -0.14 (-0.43, 0.15) | 0.07 (-0.35, 0.48) | 0.43 |
| Percent Change, % | 1.38 (-0.13, 2.88) | 2.01 (-0.11, 4.12) | 0.64 | 1.21 (-0.27, 2.68) | 1.77 (-0.31, 3.85) | 0.66 |
| E3/E4, E4/E4 |  |  |  |  |  |  |
| Absolute Change, pg/mL | -0.35 (-0.95, 0.24) | 0.69 (-0.14, 1.52) | 0.0441 | -0.42 (-1.01, 0.18) | 0.30 (-0.52, 1.13) | 0.17 |
| Percent Change, % | -1.39 (-4.33, 1.55) | 2.60 (-1.51, 6.71) | 0.12 | -0.73 (-3.67, 2.21) | 2.68 (-1.39, 6.75) | 0.18 |
| E3/E4 |  |  |  |  |  |  |
| Absolute Change, pg/mL | -0.25 (-0.87, 0.37) | 0.66 (-0.21, 1.53) | 0.10 | -0.35 (-0.97, 0.27) | 0.32 (-0.54, 1.18) | 0.21 |
| Percent Change, % | -0.82 (-3.92, 2.28) | 2.33 (-2.02, 6.68) | 0.25 | -0.41 (-3.51, 2.69) | 2.67 (-1.65, 6.99) | 0.25 |
| E4/E4 |  |  |  |  |  |  |
| Absolute Change, pg/mL | -2.06 (-3.70, -0.42) | 0.75 (-1.52, 3.01) | 0.0497 | -1.96 (-4.07, 0.14) | -0.23 (-2.96, 2.50) | 0.34 |
| Percent Change, % | -10.39 (-17.49, -3.30) | 3.08 (-6.70, 12.85) | 0.0288 | -10.51 (-18.82, -2.19) | 3.16 (-7.64, 13.97) | 0.06 |

Note: values in table are mean (95% CI)

*Results reflect adjustment for mean-centered baseline p-tau181 and mean-centered age

**eTable 8. Correlations Between End-of-Study Obicetrapib Concentration, Time-Averaged Achieved Lipid and Lipoprotein Concentrations, and Absolute and Percent Change in p-tau217 Among E4/E4 Patients**

|  | p-tau Absolute Change | p-tau Percent Change |
| --- | --- | --- |
| Obicetrapib Concentration | r = -0.64  p=0.0002 | r = -0.61  p=0.0005 |
| LDL-C | r = 0.36  p=0.0524 | r = 0.31  p=0.10 |
| HDL-C | r = -0.39  p = 0.0341 | r = -0.34  p = 0.07 |
| Lipoprotein(a) | r = 0.38  p = 0.0426 | r = 0.38  p=0.0447 |
| ApoB | r = 0.54  p = 0.0024 | r = 0.52  p=0.0041 |
| ApoA1 | r = -0.22  p=0.26 | r = -0.16  p=0.42 |

Note: time-averaged achieved lipids and lipoproteins were calculated with all available values from baseline to end of study using the linear trapezoidal rule and observed assessment times relative to baseline.
